# Metformin Reverses *tmexCD1-toprJ1*- and *tet*(A)-Mediated High-Level Tigecycline Resistance in *K. pneumoniae*

**DOI:** 10.3390/antibiotics11020162

**Published:** 2022-01-27

**Authors:** Xia Xiao, Quanmin Huan, Yanhu Huang, Yuan Liu, Ruichao Li, Xilan Xu, Zhiqiang Wang

**Affiliations:** 1 College of Veterinary Medicine, Yangzhou University, Yangzhou 225009, China; xiaoxia@yzu.edu.cn (X.X.); huanquanmin@126.com (Q.H.); yuqi29595585@163.com (Y.H.); liuyuan2018@yzu.edu.cn (Y.L.); rchl88@yeah.net (R.L.); 2 Jiangsu Co-Innovation Center for Prevention and Control of Important Animal Infectious Diseases and Zoonoses, Yangzhou 225009, China; 3 Institute of Comparative Medicine, Yangzhou University, Yangzhou 225009, China; 4 Pizhou Animal Health Supervision Institute, Xuzhou 320300, China; xiaxian6629285@163.com; 5 Institutes of Agricultural Science and Technology Development, Yangzhou 225009, China

**Keywords:** tigcycline, metformin, *tmexCD1-toprJ1*, *tet*(A), synergy

## Abstract

Tigecycline (TIG) is one of the last effective options against multidrug resistance bacteria. Recently, the RND (resistance–nodulation–division) efflux pump gene cluster, *tmexCD1-toprJ1*, and the tetracycline-efflux pump *tet*(A) mutation were reported to mediate high level resistance to TIG in clinically important pathogens, weakening the efficacy of TIG. In this study, we report the potent synergistic effect of the antidiabetic drug metformin in combination with TIG against *tet*(A) mutant and *tmexCD1-toprJ1* positive *K. pneumoniae*. The fractional inhibitory concentration index (FICI) of TIG and metformin were less than 0.05 for all the tested isolates. The time–kill curve assay showed that the combination of TIG and metformin exhibited much better antimicrobial effect than TIG alone. The synergistic effect was also confirmed in vivo using a well-studied *Galleria mellonella* larvae model. Mechanistic studies demonstrated that metformin disrupted the important component of proton motive force, the electric potential (Δψ) and the function of efflux pump, thereby increasing the intracellular concentration of TIG. This finding revealed that metformin might be a possible adjuvant of TIG for combating with superbugs carrying the *tet*(A) mutant and *tmexCD1-toprJ1* genes.

## 1. Introduction

Antimicrobial resistance (AMR) is a clear and present crisis to “one health”. Right now, at least 700,000 people succumb to AMR every year [1]. If no intervening measures are made, by 2050, 10 million patients would die and a cumulative 100 trillion dollars would be generated as a result of AMR [2]. In the last few years, plasmid-mediated resistance genes encoding New Delhi metallo-beta-lactamase (NDM) [3] and phosphoethanolamine transferase enzyme (*mcr-1*) [4] that mediate resistance to the clinical last-resort treatments (carbapenems, colistin) were identified and disseminated rapidly worldwide, immensely limited the therapeutic options in clinical practice.

Tigecycline (TIG) is a semi-synthetic parenteral glycylcycline [5]. It not only performs antibacterial effect against tetracycline resistance bacteria, but also shows excellent antibacterial effect against NDM or MCR positive bacteria [6]. TIG has been used in the clinical industry since 2005 [5], and few resistance mechanisms had emerged by 2019. However, recently, a series of resistance genes such as the *tet*(X3/X4) [7,8], the RND (resistance–nodulation–division) efflux pump gene cluster, *tmexCD1-toprJ1* [9], and the tetracycline-efflux pump *tet*(A) mutation [10,11] were reported to mediate high-level resistance to tigecycline in clinically important pathogens, weakening the efficacy of TIG. Worse, a decline in the development of new antibiotics since the 1970s accompanied this AMR crisis [12,13], threatening the convenient therapeutic options in the post-antibiotic era. Therefore, it is urgent to rescue the efficiency of TIG against *tet*(X3/X4), *tmexCD1-toprJ1* and *tet*(A) mutant positive pathogens.

The antibiotic adjuvant strategy is a cost-effective and promising approach to extend the lifespan of existing antibiotics through inhibiting bacterial resistance or enhancing antibiotic killing [14]. It was reported that the non-steroidal anti-inflammatory drug benzydamine reversed *tmexCD-toprJ*-mediated TIG resistance [15]. In our previous study, we found that the antidiabetic drug metformin showed no synergy effect with TIG against *tet*(X3/X4)-positive bacteria, but exhibited the potentiation effect on doxycycline and minocycline against MDR Gram-positive and -negative pathogens through disrupting membrane potential as well as outer membrane permeabilization, and inhibiting the functions of efflux pump [16]. As both the *tmexCD1-toprJ1* and *tet*(A) mutant mediate TIG resistance via efflux pump, we hypothesize whether the metformin could restore the efficacy of TIG against *tmexCD1-toprJ1* and *tet*(A) mutant positive *Klebsiella pneumoniae* (*K. pneumoniae*).

In this study, we sought to extend the synergistic activity of metformin in combination with TIG against MDR *K. pneumoniae* harbouring *tmexCD1-toprJ1* or *tet*(A) mutant. We found that metformin significantly potentiated the antibacterial activity of TIG against tmexCD-toprJ-and *tet* (A)- bearing bacteria, both in vitro and in the *Galleria mellonella* infection model. The potentiation of metformin to TIG is attributed to the dysfunction of the efflux pump and increasing the intracellular accumulation of TIG. Our results demonstrate that the antidiabetic drug metformin is a potent antibacterial adjuvant in conjunction with TIG for the treatment of infection caused by *tet*(A) mutant and *tmexCD1-toprJ1* positive *K. pneumoniae*.

## 2. Materials and Methods

### 2.1. Bacteria and Reagents

Two *tmexCD1-toprJ1* positive *K. pneumoniae* (RGT9-1, RGF15-2-1) and one *tet*(A) mutant positive *K. pneumoniae* RGF131 were isolated from swine faeces in 2020 by our lab. All the isolates mediate high level resistance to tigecycline (TIG) with the MIC value over than 16 µg/mL. The TIG, ciprofloxacin, meropenem, gentamicin, and metformin were purchased from Yuanye Biological Technology Company (Shanghai, China). Other fluorescence probes and reagents were purchased from Sigma-Aldrich (Saint Louis, MO, USA).

### 2.2. MIC Determination

The minimum inhibitory concentration (MIC) of TIG, ciprofloxacin, gentamicin, and metformin against *K. pneumoniae* RGT9-1, RGF15-2-1, and RGF131 were determined by the broth micro-dilution method according to the guidance of the Clinical and Laboratory Standards Institute (CLSI) and interpreted in accordance with the CLSI standard [17]. *E. coli* ATCC 25922 was used as the quality control strain.

### 2.3. Checker Board Assay

Synergistic activity between TIG or other antibiotics (ciprofloxacin, gentamicin, and meropenem) and metformin were measured by the well-studied checkerboard assay [18]. Briefly, 100µL MHB was dispensed into a 96-well micro-titer plate, and then the metformin and TIG (or ciprofloxacin, gentamicin, meropenem) were serially diluted seven times to reach an 8 × 8 matrix. Then, 100 µL fresh bacterial suspensions at a concentration of 10^6^ cfu/mL were added. The mixture was incubated at 37 °C for 18 h, and then the optical density (OD) value at 600 nm was measured by the Microplate reader (Tecan, Männedorf, Switzerland). The synergistic activity was interpreted with the fractional inhibitory concentrations index (FICI), that was calculated according to the following formula: FICI = FICA + FICB = MICAB/MICA + MICBA/MICB [18]. The FICA and FICB are the FIC index of drug A and B, respectively; MICA and MICB are the MIC of drug A and B, respectively; and MICAB and MICBA are the MIC of one drug in combination with another. Synergism was defined when the FICI is equal to or less than 0.5.

### 2.4. Time–Killing Curve

Overnight culture of each isolate was diluted 1:10,000 into fresh MH broth and incubated for 6 h at 37 °C under continuous shaking (200 rpm). Then, the culture was treated with TIG or metformin alone or their combination for 24 h. At the time points 0, 4, 8, 12, and 24 h, bacterial numbers were calculated with plate colony counting method. The concentrations of 16 µg/mL TIG and 50 µg/mL metformin was used for *K. pneumoniae* RGF131 while 32 µg/mL TIG and 25 µg/mL metformin was used for *K. pneumoniae* RGF15-2-1 and RGT9-1. MH broth with PBS were used as a negative control. Each experiment was performed with three biological replicates.

### 2.5. The Proton Motive Force Assay

The important component of proton motive force (PMF), the electric potential (Δψ) of all the three isolates treated by metformin was measured with fluorescence probe 3,3′-dipropylthiadicarbocyanine iodide (DiSC3(5)) as described in our previous study [15]. Bacterial cells were washed and resuspended to obtain an OD600 of 0.5 with PBS. Additionally, then DiSC3(5) (Aladdin, Shanghai, China) was added at a final concentration of 5 µM. After 30 min incubation, 180µL samples were transferred in a black-walled plate and fluorescence was measured immediately in an Infinite M200 Microplate reader (Tecan) with excitation wavelength at 622 nm and emission wavelength at 670 nm with an interval of 2 min for 34 min. Metformin was added at the time point of 4 min with a final concentration of 10 and 20 µg/mL.

### 2.6. Efflux Pump Assay

The effect of metformin on the function of *tmexCD1-toprJ1*- and *tet*(A)-mediated efflux pumps was evaluated with the fluorescence dye Rhodamine B [19]. The same cells as in the time–killing curve assay were washed with PBS three times and resuspended with PBS to attain a final OD of 0.5. A final concentration of Rhodamine B (5 µM for *K. pneumoniae* RGF15-2-1, RGT9-1 and 50 µM for *K. pneumoniae* RGF131) was added. Then, the mixture was cultured for 30 min at 37 °C under continuous shaking (200 rpm). The extra Rhodamine B were washed with PBS three times, then the pellets were re-suspended in PBS containing 1% glucose. Metformin at a final concentration of 25, 50, and 100 mg/mL was then added and incubated at 37 °C for 30 min. Finally, the bacteria were centrifuged at 4000× *g* for 10 min and then Rhodamine B efflux from the cells was monitored with the excitation wavelength at 540 nm and emission wavelength at 625 nm using Infinite M200 Microplate reader.

### 2.7. Tigecycline Intracellar Accumulation Analysis

The well-studied high-performance liquid chromatography-tandem mass spectrometry (HPLC-MS/MS) method for the determination of TIG was used to determine the accumulation of TIG in *K. pneumoniae* RGF15-2-1 and *K. pneumoniae* RGF131 [20]. Briefly, 1.0 mL of an overnight culture of each isolate was diluted into 100 mL fresh Luria Bertani (LB) broth and grown at 37 °C under continuous shaking (200 rpm) to an OD600 of 0.5. Then, bacteria cells were pelleted and diluted to 10^10^ cfu/mL with PBS. TIG at MIC concentration together with varying metformin were added, then samples were incubated at 37 °C under continuous shaking (200 rpm) for 15 min. The extracellular drug was discarded by centrifuging at 12,000× *g* for 10 min. The pretreatment method for drug extraction was identical with our previous report [16]. To lyse the cells, an aliquot of 500 µL water was added to the pellet, and then subjected to three freeze–thaw cycles in liquid nitrogen followed by water bath at 55 °C. The supernatant was collected via centrifugation at 12,000× *g* for 10 min. Additionally, another 500 µL acetonitrile were added to the pellet to further extract the TIG and pelleted again. The supernatants were combined and filtered with a 0.22 µm filter membrane.

Finally, the supernatants were detected by an Agilent 1260 Infinity HPLC system combined with AB SCIEX QTRAP 6500 mass spectrometer (ABSciex, Foster City, CA, USA). The TIG was separated on a C18 column. The mobile phase was a solution of Millipore water (0.2% *v*/*v* formic acid) (A) and ACN (B) with a gradient elution as follows: 0–0.5 min, 90% A; 0.5–1.5 min, 90–10% A; 1.5–5.0 min, 10% A; 5.0–5.5 min, 10–90% A; and 5.5–7.0 min, 90% A. The quantification detection of TIG was analysed by multiple reaction monitoring (MRM) with positive electrospray ionization using the m/z 586.4 → 513.3 transition.

### 2.8. Galleria Mellonella Infection Model

*Galleria mellonella* larvae was supplied by Huiyude Biotech Company (Tianjin, China). They were divided into 12 groups (*n* = 10 per group). All larvae were infected with 10^7^ CFUs *K. pneumoniae* RGT9-1, RGF15-2-1 or RGF131 suspension, respectively. At 1 h post-infection, infected larvae were treated with PBS, TIG (50 mg/kg b.w.), metformin (50 mg/kg b.w.), or the combination of TIG with metformin (25 + 25 mg/kg b.w.). The survival rates of each group were recorded for 5 days.

### 2.9. Data Analysis

All data are expressed as mean ± SD from three biological replicates. Statistical significance was determined using Graphpad Prism 7.0 software with unpaired Student’s *t*-test or non-parametric one-way ANOVA.

## 3. Results

### 3.1. The In Vitro Synergistic Activity of Tigecycline and Metformin against tmexCD1-toprJ1 and tet(A) Mutant Positive Strains

The MIC results of antibiotics and metformin were determined using the CLSI recommended broth micro-dilution method. The results are shown in Table 1. Both the *tmexCD1-toprJ1* positive strains (RGF15-2-1 and RGT9-1) exhibited high resistance to TIG, tetracycline, ciprofloxacin, and gentamicin, but were susceptible to meropenem. The *tet*(A) mutant isolate was resistance to TIG and tetracycline and susceptible to meropenem. The MIC of metformin against all the three isolates were 50 mg/mL.

**Figure 1:**
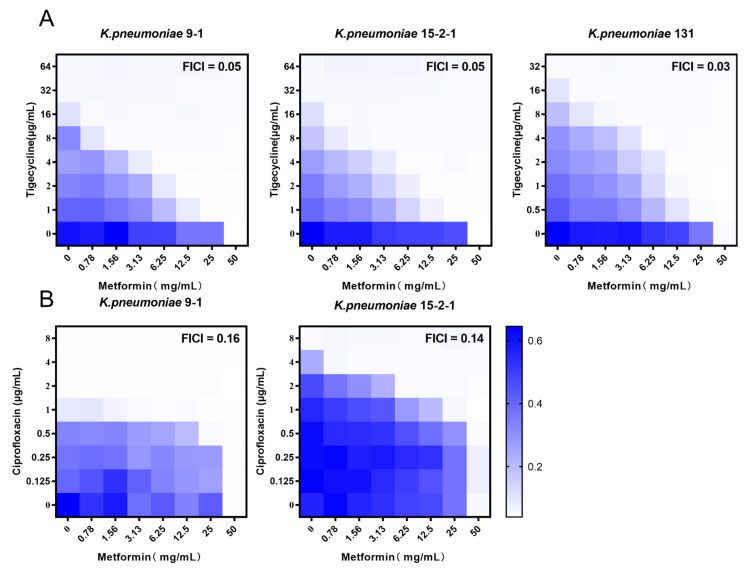
Synergistic activity of tigecycline and metformin against *K. pneumoniae* harbouring *tmexCD1-toprJ1* or *tet*(A) mutation. (**A**) Chequerboard broth microdilution assays between metformin and tigecycline against *K. pneumoniae*. (**B**) Chequerboard broth microdilution assays between metformin and ciprofloxacin against *K. pneumoniae*. Dark-blue regions represent higher cell density and lower inhibition rate of combinational treatment. Data represent the mean OD (600 nm) of two biological replicates. Synergy is defined as an FIC index of ≤0.5.

As metformin exhibits the potentiation effect on doxycycline and minocycline through inhibiting the functions of efflux pump, we tested the synergistic activity of metformin and TIG in fighting with *tmexCD1-toprJ1* and *tet*(A) mutant positive strains with checkboard assays. Notably, obvious synergism was observed for the combination against all three isolates with the FICI of 0.047, 0.047, and 0.030 in *K. pneumoniae* RGT9-1, RGF15-2-1, and RGF131, respectively (Figure 1A). To further test whether this synergy effect was TIG-specific, the synergistic effects of metformin and other antibacterial (meropenem, ciprofloxacin, and gentamicin) were assessed through checkboard assay. Metformin potentiated the antibacterial effect of ciprofloxacin against *tmexCD1-toprJ1* positive *K. pneumoniae* in fighting with RGF15-2-1 and RGT9-1 with the FICI of 0.14 and 0.16 (Figure 1B). However, an antagonistic effect was observed in the combination of ciprofloxacin and metformin in fighting with *tet*(A) mutant positive *K. pneumoniae* RGF131 (data not shown). As the resistance to TIG and ciprofloxacin was mediated by *tmexCD1-toprJ1* in RGF15-2-1 and RGT9-1 and the *tet*(A) mutant only mediated resistance to TIG and other tetracyclines in RGF131, we hypothesize metformin inhibited the function of the efflux pump. The antagonism effect was also observed in the combination of metformin and meropenem or gentamicin against RGF15-2-1 and RGT9-1 (Figure 1). Though *tmexCD1-toprJ1* mediated resistance to aminoglycoside, there was other resistance gene playing a role in gentamicin resistance; for example, the aminoglycoside adenyltransferase (*aadA2*) [21]. The results indicate that metformin may potentiate the effect of TIG through inhibit the *tmexCD1-toprJ1* and *tet*(A) efflux pump.

**Figure 2:**
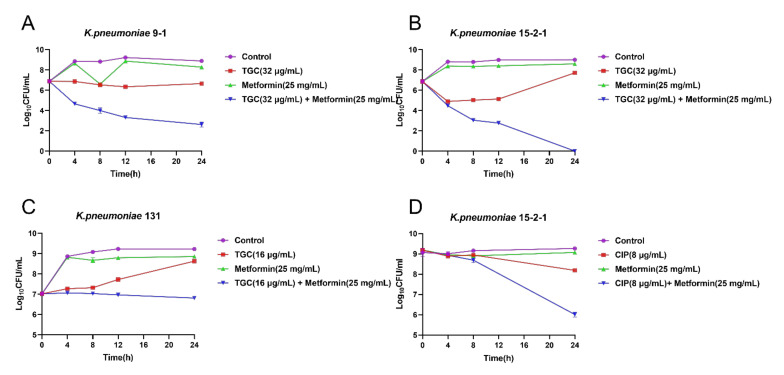
Time–killing curves of the combination of tigecycline and metformin against (**A**) *K. pneumonia* 9-1 harbouring *tmexCD1-toprJ1*; (**B**) *K. pneumoniae* 15-2-1harboring *tmexCD1-toprJ1*; (**C**) *K. pneumoniae* 131 harbouring *tet*(A) mutation; and (**D**) Time–killing of the combination of ciprofloxacin and metformin against *K. pneumonia* 15-2-1 harbouring *tmexCD1-toprJ1*.

To further investigate the synergistic effect of TIG and metformin, the time–kill curves were performed for TIG or metformin monotherapy and their combination. We found that either 32 µg/mL TIG (corresponding to MIC) or 25 mg/mL metformin (corresponding to 1/2 MIC) alone displayed no effect on bactericidal effect. In the contrast, the combination of 32 µg/mL TIG and 25 mg/mL metformin showed obvious bactericidal activities against both RGT9-1 and RGF15-2-1 (Figure 2A,B). The combination effect of TIG and metformin also showed superiority in anti-bacteria effect compared to single drug treatment in the case of *tet*(A) mutant isolate RGF131 (Figure 2C). Furthermore, the combination of 8 µg/mL ciprofloxacin and 25 mg/mL metformin showed obvious bactericidal activities against RGF15-2-1 while the single drug cannot even inhibit the growth of RGF15-2-1 (Figure 2D). This results indicated that metformin possesses universal synergistic effect with antibacterial which was inactivated by the *tmexCD1-toprJ1* or *tet*(A) efflux pump.

### 3.2. Metformin Deprives the Function of Efflux Pump and Facilitate the Intracellular Accumulation of Tigecycline

**Figure 3:**
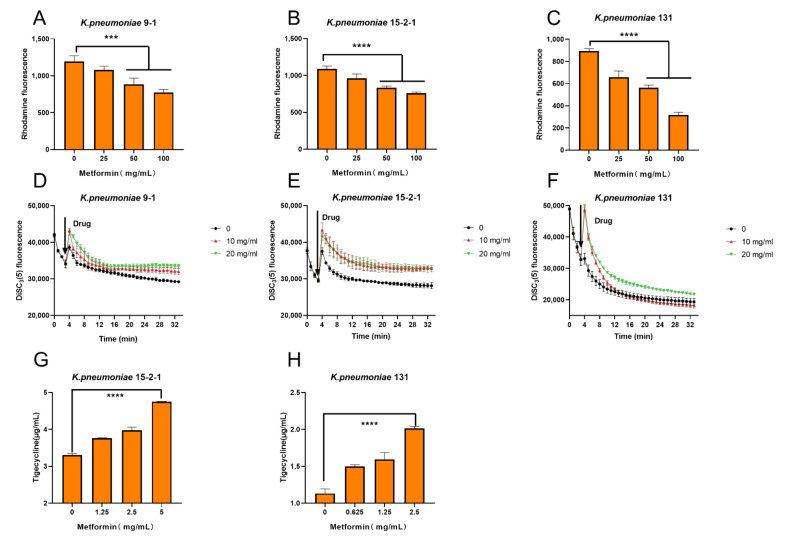
Tigecycline–metformin combination exerts synergy by dissipating electric potential (Δψ), destroying the capability of the efflux pump and increase the intracellular concentrations of tigecycline. (**A**–C) Function of efflux pump of (**A**) *K. pneumonia* 9-1 harbouring *tmexCD1-toprJ1*; (**B**) *K. pneumoniae* 15-2-1harboring *tmexCD1-toprJ1*; and (**C**) *K. pneumoniae* harbouring *tet*(A) mutation after exposure to varying concentrations of metformin, measured by the fluorescence dye Rhodamine. (**D**–**F**) Membrane potential changes in (**D**) *K. pneumonia* 9-1 harbouring *tmexCD1-toprJ1*; (**E**) *K. pneumoniae* 15-2-1 harbouring *tmexCD1-toprJ1*; and (**F**) *K. pneumoniae* harbouring *tet*(A) mutation upon exposure to metformin, probed by potentiometric fluorophore DiSC3(5). (**G**,**H**) Intracellular accumulation of tigecycline in cells treated with metformin determined by HPLC-MS/MS analysis. Initial concentration of tigecycline was 32 µg/mL. All data are expressed as mean ± SD from three biological replicates and *p* values were determined by non-parametric one-way ANOV A (*** *p* < 0.001, **** *p* < 0.0001).

To elucidate the possible mechanisms of the synergy effect between metformin and TIG, the effect of metformin on the *tmexCD1-toprJ1* and *tet*(A) efflux pump function was investigated. The capability of the *tmexCD1-toprJ1* and *tet*(A) efflux pumps under the treatment of different concentrations of metformin was assessed with a rhodamine-based assay [15]. The effluxes of rhodamine was reduced in a dose-dependent way at the presence of metformin in all the three isolates (Figure 3A–C).

As the proton motive force (PMF) is essential for the function of efflux pump, we also tested the important component of PMF, the electric potential (Δψ) of all the three isolates treated by metformin with 3,3′-dipropylthiadicarbocyanine iodide (DiSC3(5)) probe. As expected, the addition of metformin at 10 and 20 mg/mL significantly increased the fluorescence. The increased fluorescence indicated the release of DiSC3(5) from the cytoplasmic membrane to the extracellular milieu due to the disruption of Δψ. Thus, the Δψ was disrupted under metformin treatment (Figure 3D–F), as was the PMF.

The intracellular concentration of TIG in all the three isolates after treatment with a series concentration of metformin was measured by HPLC-MS/MS. The concentrations of TIG in bacteria were significant increased in a dose-dependent way at the presence of metformin (Figure 3G,H). The results indicated that TIG was markedly accumulated in bacteria cells under the treatment of metformin.

### 3.3. Metformin Potentiates In Vivo Efficacy of Tigecycline Using Galleria Mellonella Infection Model

**Figure 4:**
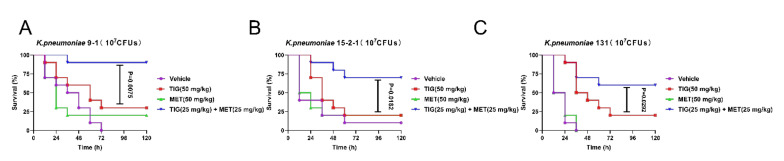
Metformin effectively improves tigecycline efficacy in *Galleria mellonella* infection model. Survival rates of *Galleria mellonella* larvae (*n* = 10 per group) infected by (**A**) *K. pneumoniae* 9-1 harbouring *tmexCD1-toprJ1*; (**B**) *K. pneumoniae* 15-2-1 harbouring *tmexCD1-toprJ1*; and (**C**) *K. pneumoniae* harbouring *tet*(A) mutation after treatment with PBS as vehicle, a single dose of metformin (MET, 50 mg/kg), tigecycline (TIG, 50 mg/kg), or their combination with concentration of 25 mg/kg, respectively. *p* values were determined by log-rank (Mantel–Cox) test.

After confirming the in vitro synergistic activity of TIG and metformin against *tmexCD1-toprJ1* and *tet*(*A*) mutant positive strains, we further assessed whether these synergy effects could be observed in vivo. To confirm this, a well-studied preclinical infection model (*Galleria mellonella* larvae infection models) was constructed with all the three isolates. The larvae in blank control all died in 72 h, except the strain of RGF15-2-1. The survival rates in TIG or metformin monotherapy groups were below 25% for all the isolates. In contrast, the survival rates in combination groups were significantly increased with the *p* values of 0.002, 0.010, and 0.029 compared to TIG monotherapy group for infection of *K. pneumoniae* RGT9-1, RGF15-2-1, or RGF131, respectively (Figure 4A–C). It should be stressed here that the dose in combination group was just half that in monotherapy groups. The in vivo results demonstrated the adjuvant potential of metformin with TIG in fight with infectious diseases caused by *tmexCD1-toprJ1* and *tet*(A) mutant positive pathogens. 

## 4. Discussion

TIG was the last antibiotic choice in fighting carbapenems and colistin resistance Gram-negative pathogen infections. However, with the identification and spreading of the genetic elements *tet*(X3/X4), *tmexCD1-toprJ1*, and *tet*(A) mutant mediating high levels of TIG resistance, the effect of TIG was much weakened [8,9,11]. Fortunately, the adjuvant strategy, which restores antibiotic efficacy against MDR pathogen infection, was prosperous in recent years. For example, thioridazine restores the antibacterial effect of oxacillin against MRSA [22]; a synergy effect was observed between Phenothiazines and Oxacillin against MRSA [23]; and anti-HIV agent azidothymidine decreases *tet*(X) mediated bacterial resistance to tigecycline in *Escherichia coli* (*E. coli*) [24]. These exciting results inspire us to find more potential adjuvants of the last restored drug TIG against super bugs. In our previous study, we found that an FDA-approved hypoglycemic drug metformin potentiates tetracyclines (except TIG) against multiple *tet*(A) positive pathogens including *S. aureus*, vancomycin-resistant *enterococci*, *E. coli*, and *S. enteritidis* [16]. We speculated that the low level of TIG resistance mediated by *tet*(A) may account for the no synergy effect between metformin and TIG. Thus, in this study, we sought to extend the synergistic activity of metformin in combination with TIG against *K. pneumoniae* harbouring *tmexCD1-toprJ1* or *tet*(A) mutant. Interestingly, synergistic activity was observed between metformin and TIG against *tet*(A) mutant positive *K. pneumoniae* RGF131 with the FICI of 0.03. It is worth noting that the *tet*(A) mutant mediated high level resistance to TIG with the MIC value of 32 µg/mL in *K. pneumonia* RGF131. In the meantime, metformin significantly potentiated the antibacterial activity of TIG against *tmexCD1-toprJ1* bearing bacteria, which mediated resistance against multiple kinds of antimicrobials such as tetracyclines and fluoroquinolones. On the other hand, synergy was also observed between metformin and ciprofloxacin against *tmexCD1-toprJ1* bearing bacteria, indicating metformin was a universal adjuvant against *tmexCD1-toprJ1* positive bacteria. The time–kill curve study not only verified the synergistic activity of metformin in combination with TIG or ciprofloxacin, but also displayed a time-dependent bactericidal activity of the combinations, highlighting the potency of the drug combination. The in vivo synergism effect of metformin and TIG against all three isolates were assessed and verified using a well-studied *Galleria mellonella* infection model. It is a pity that the adjuvant therapy effect of this combination was not tested in a rodent disease model in this study. As the adjuvant potential of metformin with doxycycline was obvious in a mouse peritonitis infection model infected with *E. coli* B2, the in vivo synergism effect of metformin and TIG was expected in rodent disease model. However, further study is needed to confirm it.

It was reported in our previous study that metformin potentiated doxycycline antibacterial effects through disrupting membrane potential (∆ψ) [16]. Thus, the ∆ψ was assessed in the presence of metformin. As expected, the ∆ψ was significantly disrupted. It was reported that the ∆ψ is an important component of PMF [25]. Additionally, the PMF was essential for the function of efflux pump [26]. The dissemination of PMF might affect the capability of the efflux pump. Interestingly, the activity of the efflux function of *tmexCD1-toprJ1* and *tet*(A) mutant was weakened in a dose-dependent manner by metformin in the rhodamine assay. Similarly, the intracellular concentrations of TIG were markedly increased under the action of metformin. As TIG only exhibits antibacterial activity when a sufficient drug enters into bacteria cells and binds to the 30S ribosomal subunit [27], intracellular TIG concentration is important for the antibacterial effects of TIG. Metformin disrupted the PMF, hence blocking the *tmexCD1-toprJ1* and *tet*(A) efflux pumps and promoting the accumulation of TIG in cells. Thus, the potentiation of metformin to TIG is attributed to the dysfunction of the efflux pump and increasing the intracellular accumulation of TIG. These findings demonstrate that antidiabetic drug metformin is a potent antibacterial adjuvant in conjunction with TIG for the treatment of infection caused by *tet*(A) mutant and *tmexCD1-toprJ1* positive *K. pneumoniae*.

## Figures and Tables

**Table 1:** Basic information and susceptibility profiles of *K. pneumoniae* harbouring *tmexCD1-toprJ1* or *tet*(A) mutation.

Strain	Resistance Gene	Sources	MIC (;µg/mL)										
			GEN	CFF	CIP	ENR	TET	TGC	DOX	MET	MER	CL	KAN
RGT9-1	*tmexCD1-toprJ1,oqxAB, QnrB4, QnrS1, aac(3)-IId, aac(6’)Ib-cr, aadA16, aadA2*	Swine Faeces	128	8	8	8	>128	64	64	50 mg	≤0.25	≤0.25	>256
RGF15-2-1	*tmexCD1-toprJ1,oqxAB, QnrB4, QnrS1, aac(3)-IId, aac(6’)Ib-cr, aadA16, aadA2*	Swine Faeces	128	16	8	8	>128	64	64	50 mg	≤0.25	≤0.25	>256
RGF-131	*tet*(A), *QnrS1*, *aac(3)-IId*, *aac(6’)Ib-cr*, *aadA16*, *aadA1*, bla*_CTX-M-55_*, bla*_SHV-11_*, bla*_SHV-1_*	Swine Faeces	64	4	4	4	>128	32	64	50 mg	≤0.25	≤0.25	>256

GEN, gentamicin; CFF, ceftiofur; CIP, ciprofloxacin; ENR, enrofloxacin; TET, tetracycline; TGC, tigecycline; DOX, doxycycline; MET, metformin; MER, meropenem; CL, colistin; and KAN, kanamycin.

## Data Availability

Data is contained within the article.
